# Photomechanochemistry: harnessing mechanical forces to enhance photochemical reactions

**DOI:** 10.3762/bjoc.21.33

**Published:** 2025-03-03

**Authors:** Francesco Mele, Ana Maria Constantin, Andrea Porcheddu, Raimondo Maggi, Giovanni Maestri, Nicola Della Ca’, Luca Capaldo

**Affiliations:** 1 SynCat Lab, Department of Chemistry, Life Sciences and Environmental Sustainability, University of Parma, Parco Area delle Scienze 17/A, 43124 Parma, Italy; 2 Dipartimento di Scienze Chimiche e Geologiche, Università degli Studi di Cagliari, Cittadella Universitaria, SS554 bivio per Sestu, 09042-Monserrato (CA), Italy; 3 CIRCC (Interuniversity Consortium Chemical Reactivity and Catalysis), via Celso Ulpiani 27, 70126 Bari, Italy; 4 Department of Chemistry, Life Sciences and Environmental Sustainability, University of Parma, Parco Area delle Scienze 17/A, 43124 Parma, Italy

**Keywords:** light-mediated synthesis, mechanochemistry, photomechanochemistry

## Abstract

Photomechanochemistry, i.e., the merger of light energy and mechanical forces, is emerging as a new trend in organic synthesis, enabling unique reactivities of fleeting excited states under solvent-minimized conditions. Despite its transformative potential, the field faces significant technological challenges that must be addressed to unlock its full capabilities. In this Perspective, we analyze selected examples to showcase the available technologies to combine light and mechanical forces, including manual grinding, vortex and shaker mixing, rod milling, and ball milling. By examining the advantages and limitations of each approach, we aim to provide an overview of the current state of synthetic photomechanochemistry to identify opportunities for future advancements in this rapidly evolving area of research.

## Introduction

Light-mediated synthetic methodologies have significantly transformed contemporary organic chemistry by enabling a broad array of previously unattainable transformations [1]. In fact, the absorption of a photon by a molecule causes the reorganization of the electron density around the atoms and unlocks unique reactivity modes [2–3]. In photochemical methods, light is directly absorbed by a functional group embedded in the substrate and can be exploited for example to cleave bonds or trigger rearrangements. Most organic molecules are colorless and, in fact, do not absorb visible light: highly energetic UV irradiation is typically needed. A milder approach is offered by photocatalytic approaches. Here, a photocatalyst is added to the reaction mixture to convert light energy into chemical potential to transform molecules. Intriguingly, photocatalysts typically absorb harmless visible light and can be chosen *ad hoc* to trigger the desired chemistry. Indeed, the photocatalyst–substrate interaction can occur via energy transfer [4–8], single-electron transfer [9–12], or hydrogen-atom transfer [13–15].

**Figure 1:**
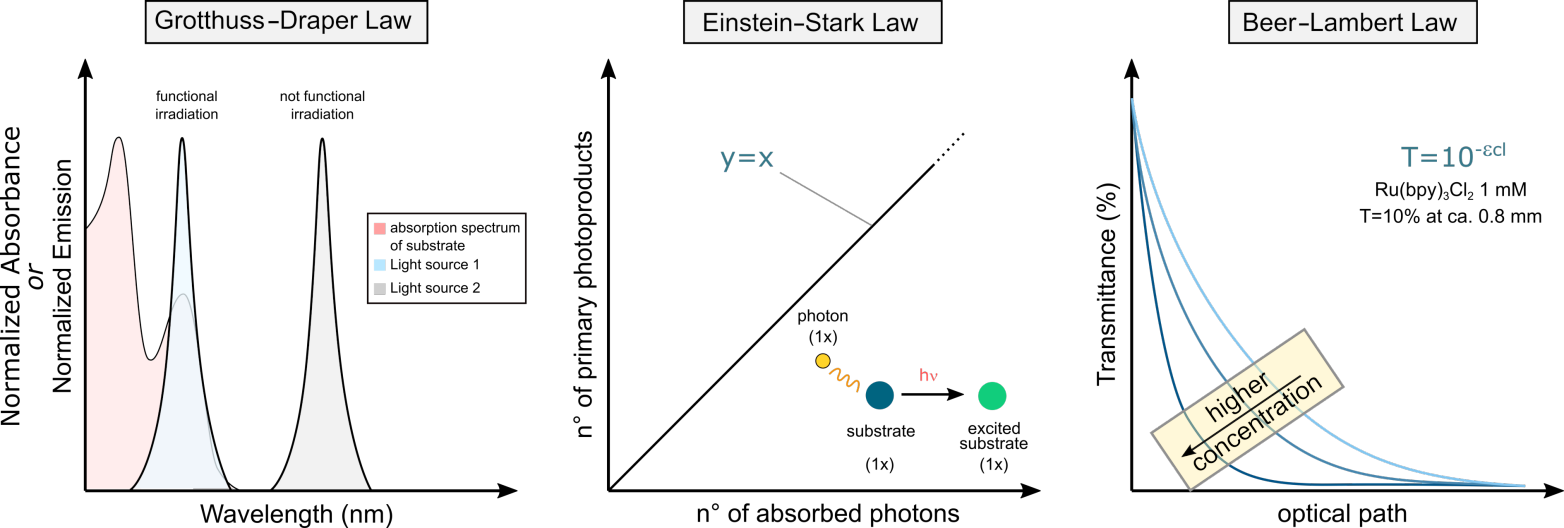
The Grotthuss–Draper, Einstein–Stark, and Beer–Lambert laws. *T*: transmittance; ε: molar attenuation coefficient; *c*: concentration; *l*: optical path.

Regardless of the mechanistic details of the activation manifold, all photochemical reactions obey two laws: the Grotthuss–Draper and the Einstein–Stark laws [16]. The Grotthuss–Draper law dictates that only absorbed light can induce photochemical transformations within a system. In his own words, “independently on its chemical nature, each body reacts more strongly to the color that is complementary to that it shows in the normal state” [17]. While this concept may appear self-evident to contemporary photochemists, it served as an early cautionary note to consistently measure the absorption spectrum of reagents and ensure that the emission spectrum of the light source used overlaps (at least to some extent) with it (Figure 1). The Stark–Einstein law, sometimes referred to as the law of photochemical equivalence, asserts that the absorption of light is a quantum process involving one photon per absorbing molecule (or atom) [18]. It should be stressed that one photon might trigger a chain of events leading to the formation of multiple molecules of product (Figure 1). The Grotthuss–Draper and Einstein–Stark laws mainly predict the feasibility of a light-mediated transformation from a conceptual standpoint.

Another fundamental law in photochemistry is the Beer–Lambert law, which describes a negative exponential correlation between the transmittance of a solution containing chromophores (i.e., a light-absorbing species) and the optical path length (Figure 1). In essence, as a beam of light traverses through a solution, photons are quickly absorbed, resulting in abrupt reduction in light intensity. Thr Beer–Lambert law imposes the most stringent constraints from a practical point of view, mainly in terms of scalability. This requires highly diluted solutions, which in turn demands large volumes of solvents, negatively impacting the sustainability of light-mediated synthesis as it transitions from academic curiosity to industrial application. Moreover, since the solvent is the reaction component with the highest concentration, competitive side-reactions such as hydrogen-atom transfer or solvolysis are often observed.

A technological solution to cope with the Beer–Lambert law was offered by flow chemistry [19–21] by employing microreactors with reduced optical paths to enhance irradiation efficiency [22–24]. Photon-limited reactions, whose efficiency is primarily constrained by the availability of photons in the reaction mixture, particularly benefit from this approach. Paradoxically, flow chemistry is a convenient technology to increase the productivity of photochemical reactions via numbering-up and sizing-up approaches [25], but it is highly dependent on solvents. In fact, high concentrations of reagents or products can lead to precipitation, causing undesired clogging of the reactor and potentially disastrous consequences for the intended transformation. It is important to mention that specific solutions for handling slurries in flow have been developed, where additional energy is applied to increase mixing such as pulsation [26–28], ultrasound energy [29], segmented flow [30], or mechanical stirring [31].

In line with the recent emphasis on Green Chemistry principles [32–33], alternatives have been developed in order to reduce the amount of organic solvents [34] required for light-mediated transformations including on- and in-water approaches [35–37], the use of supercritical CO_2_ as solvent [38], and the melting point depression strategy [39]. However, a more drastic option would be to remove the solvent: no solvent is the best solvent.

Mechanochemical synthesis, particularly through ball milling, has emerged as a powerful and sustainable technique that offers numerous advantages over traditional solution-phase methods [40–42]. By often eliminating the need for solvents, mechanochemistry reduces environmental impact and simplifies experimental procedures. Moreover, it often leads to shorter reaction times [43–44] and can overcome solubility limitations [45]. Similar to the effect of photons in light-mediated synthesis, mechanical forces generated during ball milling can induce unconventional reaction pathways [46] enabling the synthesis of novel compounds and materials. While the mechanisms underlying mechanochemical reactions are still under investigation [47–50], the increasing body of research demonstrates the potential of this technique to revolutionize synthetic chemistry.

**Figure 2:**
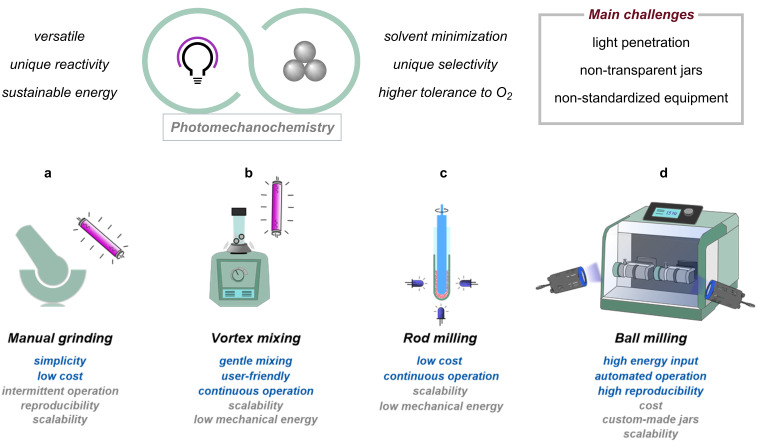
The benefits of merging photochemistry with mechanochemical setups (top). Most common setups for photomechanochemistry (bottom): a) manual grinding, b) vortex mixing, c) rod milling, and d) ball milling.

Combining photochemistry with mechanochemistry holds potential for unique opportunities for substrate activation while adopting an environmentally benign emerging technology (Figure 2, top). For example, it is well known that molecules in the solid state (or in very high concentration) often exhibit photophysical behaviors distinct from those observed in dilute solutions because of the formation of aggregates perturbing electronic transitions [51–53]. Moreover, at high concentrations, the formation of electron donor–acceptor (EDA) complexes [54] or exciplexes is expected to be favored, and the effect of mechanical forces on these is worth being thoroughly investigated. However, to benefit from these unique opportunities, the scientific community must resolve both conceptual and practical challenges. On one side, light penetration is penalized by the absence of the solvent, especially in the case of non-transparent solids. On the other side, typical reactors used for classical mechanochemical synthesis are made of non-transparent materials such as stainless steel (SS), zirconia, or polytetrafluoroethylene (PTFE), making the use of photons operationally difficult.

In this Perspective, we gathered selected examples to showcase how light energy and mechanical forces have been integrated through the years, either in a combined or sequential manner. Our goal is not to provide an exhaustive review on photomechanochemistry [34,42,55], nor a detailed explanation of solid-state photochemistry [56–57], but rather to capture a snapshot of the current technological advancement in the field. This Perspective is organized into four sections based on the adopted setup for photomechanochemistry: manual grinding, vortex mixing, rod milling methods, and ball milling (Figure 2, bottom).

One final note is about the solvent adopted: in some examples described below, minimal amounts of solvent are used to homogenize the reaction mixture. This approach is called liquid-assisted grinding (LAG) and it is commonly accepted as a mechanochemical approach [58].^.^ Throughout the text, we adopted the term photomechanochemistry to include both photochemical and photocatalytic methods [59], while others have used "mechanophotocatalysis" or "mechanophotochemistry" for greater specificity.

## Discussion

### Experimental setups for photomechanochemistry

As mentioned above, the goal of the present perspective article is to put the spotlight on the technological advancement in the emerging field of photomechanochemistry, starting from simple manual grinding to more modern ball-milling techniques.

*Manual grinding* (Figure 2a) with a pestle and mortar is the simplest and most cost-effective technique for photomechanochemistry, relying on hand-applied forces to maximize the surface area of the solids and, therefore, irradiation efficiency. UV lamps (e.g., medium-pressure Hg lamps) are typically used for irradiation, thus requiring specialized safety equipment. While it offers some control over pressure and duration, manual grinding suffers from inconsistencies due to operator variability, limited throughput (and, hence, scalability), and challenges in reproducibility. This approach is not operated in continuo: grinding and irradiation occur sequentially and not simultaneously.

*Vortex shakers* (Figure 2b), which provide circular motion for mixing, are cheap, user-friendly and available in most laboratories. However, they have limited energy input, making them less effective for high-energy reactions, and are typically used for small-scale experiments. Also in this case, the integration of light sources requires specialized setups (vide infra). Automated vortex milling for photomechanochemistry is possible.

*Rod milling* (Figure 2c) involves the use of a glass rod inserted into a transparent glass test tube containing the reaction mixture. As the rod rotates, it generates the mechanochemical forces required to drive the reaction while continuously exposing fresh surface area to light. This setup allows the reaction mixture to be irradiated efficiently using inexpensive and energy-saving light-emitting diodes (LEDs). Importantly, this apparatus can be operated in continuo, providing opportunity for limiting human intervention.

*Ball milling* (Figure 2d) stands out for its high-energy input, automated operation, and reproducibility, making it ideal for larger-scale and more force-intensive reactions. Both LED strips and LED lamps are used as light sources in combination with transparent jars in PMMA, glass, or epoxy resins. It can achieve consistent results but comes with higher costs, more complex operation, and challenges in effectively integrating light sources for photomechanochemical reactions.

### Manual grinding

Manual grinding was predominantly used in the early days of photomechanochemistry, when [2 + 2] photochemical cycloadditions were extensively studied to get insights into the impact of light energy onto crystals [60–61]. In the following, we describe selected examples with an emphasis on the role of manual grinding in improving irradiation.

**Scheme 1:**
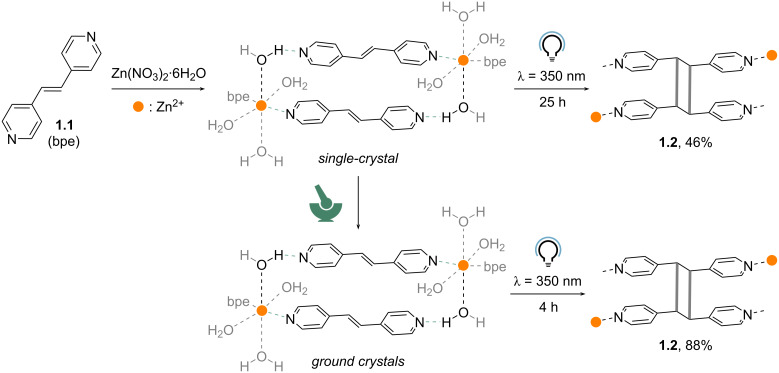
Mechanochemically triggered pedal-like motion in solid-state [2 + 2] photochemical cycloaddition for the formation of cyclobutanes [62].

Mechanochemistry is a linchpin in topochemical solid-state reactions, where the correct molecular alignment within the crystal drives the reaction. For example, in 2008, Vittal et al. [62] designed a mechanochemical strategy to align the C=C bonds of 4,4'-bipyridylethylene (bpe, **1.1**) molecules to drive efficient [2 + 2] cycloaddition and give the corresponding cyclobutanes (**1.2**). This approach relied on zinc cations to align **1.1** through the formation of hydrogen-bonded coordination complexes. Thus, when a single crystal of the [Zn(bpe)_2_(H_2_O)_4_](NO_3_)_2_·8/3H_2_O·2/3bpe complex was exposed to UV irradiation (dark blue phosphor lamps, λ = 350 nm) for 25 h, only 46% conversion to **1.2** was observed via ^1^H NMR spectroscopy (Scheme 1). During the reaction progress, the C=C bonds of bpe ligands undergo pedal-like motion prior to photodimerization [63]. For the single-crystal irradiation, the slow reactivity can be attributed to the hindered pedal motion in the single crystals, likely due to the presence of coordinative bonds on one side and hydrogen bonds on the other. Surprisingly, when the crystals were manually ground for 5 min before irradiation, the conversion to dimer **1.2** remarkably increased to 88% in only 4 h of UV irradiation time. Overall, the role of manual grinding was not only to increase the surface area exposed to light but also to allow the motions within the crystal.

**Scheme 2:**
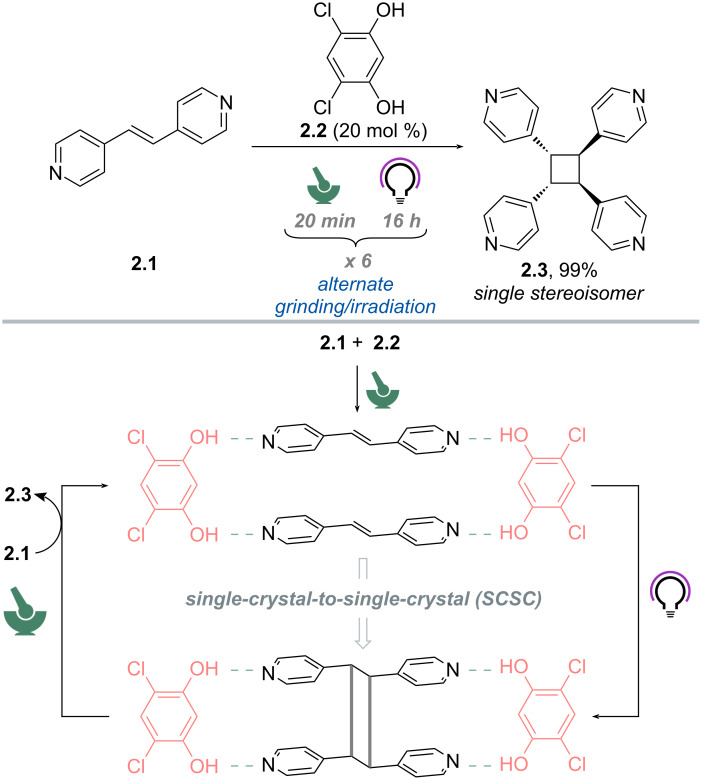
Mechanically promoted [2 + 2] photodimerization of *trans*-1,2-bis(4-pyridyl)ethylene (**2.1**) via supramolecular catalysis by 4,6-dichlororesorcinol (**2.2**) in the solid state [64].

In another instance, MacGillivray and colleagues reported the synthesis of *rctt*-tetrakis(4-pyridyl)cyclobutane (**2.3**) via [2 + 2] photodimerization of *trans*-1,2-bis(4-pyridyl)ethylene (**2.1**) via supramolecular catalysis by 4,6-dichlororesorcinol (**2.2**) in the solid state (Scheme 2) [64]. The authors noticed that single crystals of **2.1**·**2.2** (1:1 stoichiometry) produced by mortar-and-pestle grinding undergo a single-crystal-to-single-crystal (SCSC) transformation when exposed to UV light (medium-pressure Hg lamp). The cyclobutane **2.3** was obtained as a single stereoisomer and in quantitative yield after 80 h of irradiation. The role of **2.2** is to create a close-packed environment via hydrogen-bond interactions where the [2 + 2] photodimerization can occur stereoselectively. Next, the authors evaluated the possibility of using the template agent **2.2** in a sub-stoichiometric fashion (20 mol %). However, even upon prolonged exposure, the yield of **2.3** did not exceed 20% (by ^1^H NMR spectroscopy). To achieve turnover, the photoreacted solid was subjected to a second grinding and exposed again to UV irradiation for an additional 16 h, which resulted in 40% yield. Alternating grinding and irradiation, the authors managed to obtain quantitative conversion of **2.1** to **2.3**. Overall, in this work, mechanochemistry worked akin to agitation provided by stirring or heating in solution. In fact, ^1^H NMR spectroscopy, XRD and DFT analyses showed that grinding serves to dissociate the more loosely bound **2.3** from **2.2**, where a release of strain energy and formation of a more thermodynamically stable **2.1**·**2.2** complex for a subsequent turnover are favored.

**Scheme 3:**
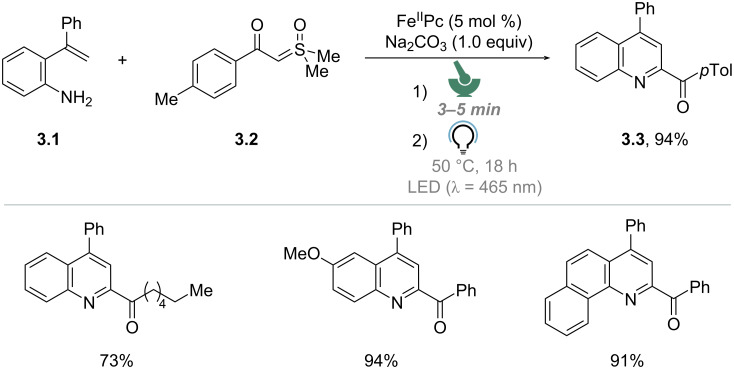
Photo-thermo-mechanosynthesis of quinolines [65].

The first preparative example of photomechanochemistry via manual grinding was reported by Wang and co-workers in 2022. They reported the photo-thermo-mechanochemical approach for the synthesis of quinolines from sulfoxonium ylides and 2-vinylanilines promoted by an iron(II) phthalocyanine (Fe^II^Pc) photocatalyst (Scheme 3) [65]. First, a mixture of 2-(1-phenylvinyl)aniline (**3.1**), sulfoxonium ylide **3.2**, and Na_2_CO_3_ was ground in a mortar at room temperature for 3–5 min. Second, the reaction mixture was transferred into a quartz tube, heated to 50 °C (heating mantle) for 18 h, while being irradiated with blue LEDs under air-equilibrated conditions. In these conditions, product **3.3** was isolated in excellent yield (94%). The reaction remained efficient across a variety of substrates, as shown in Scheme 3. The authors noted that, if the mixture was not ground before irradiation, product **3.3** was obtained in just 46% yield after isolation. Thus, the role of mechanochemistry was to achieve optimal mixing of the starting materials. The authors also noted that the reaction proceeds in the dark upon vigorous heating (120 °C). The reaction could be performed on a gram scale as well, leading to the formation of **3.3** in 92% yield.

The examples described above demonstrate that manual grinding offers simplicity and low cost for photomechanochemical reactions. However, its primary limitation lies in the manual operation and the separation of the grinding and photoreaction steps. This approach affects the reproducibility of the reaction, as it depends on the energy applied by the operator. Moreover, it is time-consuming and impractical.

### Shaking and vortex grinding

To overcome the limits of manual grinding, mechanochemists have introduced automated shaking (e.g., vortex grinding) to enable the simultaneous application of mechanical forces and photon exposure.

**Scheme 4:**
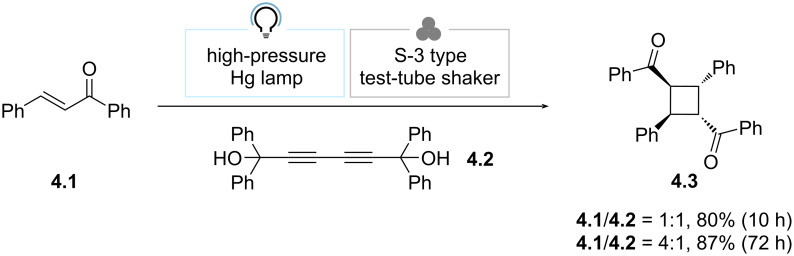
Study of the mechanically assisted [2 + 2] photodimerization of chalcone [66].

In an early example, Toda et al. discovered that shaking a 1:1 mixture of chalcone (**4.1**) and 1,1,6,6-tetraphenylhexa-2,4-diyne-1,6-diol (**4.2**) with a test-tube shaker under irradiation with a high-pressure Hg lamp, the [2 + 2] *syn*-head-to-tail dimer product **4.3** was obtained selectively in 80% yield after 10 h (Scheme 4) [66]. In detail, **4.2** works as a host to direct the photodimerization of **4.1** in a stereoselective fashion. The authors noted that, in stark contrast, the irradiation of **4.1** alone in a crystalline state yielded a mixture of isomers of **4.3** in low yield. Intriguingly, when a 4:1 ratio of **4.1** and **4.2** was used, **4.3** was obtained in 87% yield, albeit after 72 h of irradiation. This experiment proved that **4.2** can be used in a sub-stoichiometric way, in fact acting as a catalyst.

More recently, based on their previous approach via manual grinding (Scheme 2) [64], MacGillivray and co-workers adopted the vortex grinding method to enable automated and continuous mixing during irradiation, thus increasing the reproducibility and convenience of the process [67]. In particular, the authors found that the simultaneous irradiation using a low-pressure Hg lamp and grinding led to 97% ^1^H NMR yield of **2.3** in 24 h starting from **2.1** and **2.2**, which corresponds to a 4-fold improvement on reaction rates compared to manual grinding [64]. The authors proposed that continuous mechanical stress results in an increase of nucleation sites and allows catalysis to be accelerated with respect to manual grinding. Moreover, continued mechanical stress imparted by the adopted Zn-plated steel balls could also relieve any stress that builds up in the solid as a result of the photoreaction.

**Scheme 5:**
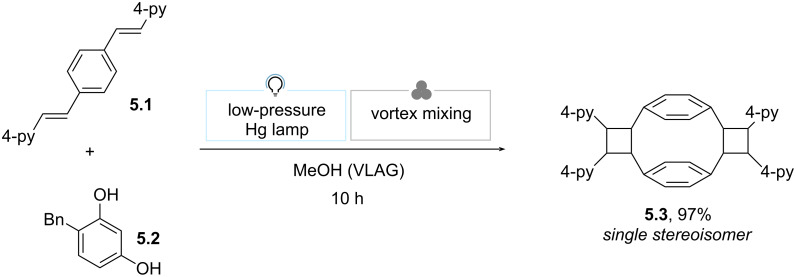
Liquid-assisted vortex grinding (LAVG) for the synthesis of [2.2]paracyclophane [68].

In 2014, the MacGillivray group resorted to liquid-assisted vortex grinding (LAVG) to broaden the applicability of vortex grinding (Scheme 5) [68]. The authors reported that when a 1:1 mixture of *p*-di[2-(4-pyridyl)ethenyl]benzene (**5.1**) and 4-benzylresorcinol (**5.2**) was ground with a mortar and pestle (1 h) and then irradiated with a low-pressure Hg lamp (60 h), [2.2]paracyclophane (**5.3**) was formed in poor yield (35% by ^1^H NMR spectroscopy). Dry vortex grinding did not improve the result. However, the addition of a LAG agent (MeOH, 50 μL) in the automated grinding step dramatically improved the outcome (quantitative conversion after 20 h of irradiation). Intriguingly, the simultaneous LAVG and irradiation enabled the formation of **5.3** in 97% yield upon reduced irradiation time (10 h).

### Rod milling

A different approach is offered by rod milling. It employs a glass rod positioned within a transparent glass test tube that contains the reaction mixture. Through the rotation of the rod, shearing forces are gently applied to facilitate the reaction, while continuously exposing fresh material to light.

**Scheme 6:**
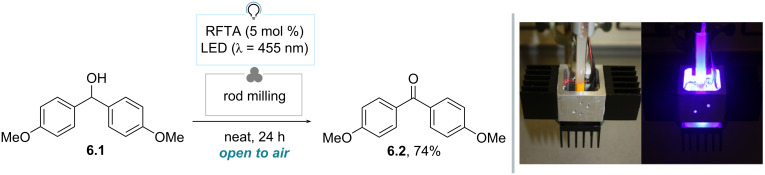
Photomechanochemical approach for the riboflavin tetraacetate-catalyzed photocatalytic oxidation of alcohols. The photos in Scheme 6 were reproduced from [69] (© 2016 M. Obst et al., published by Beilstein-Institut, distributed under the terms of the Creative Commons Attribution 4.0 International License, https://creativecommons.org/licenses/by/4.0).

In 2016, König and co-workers exploited rod milling to develop a photomechanochemical approach for the riboflavin tetraacetate (RFTA)-catalyzed photocatalytic oxidation of alcohols to the corresponding carbonyl compounds upon irradiation with five LEDs (λ = 455 nm) [69]. As an example, the authors were able to convert 4,4’-dimethoxybenzhydrol (**6.1**) to 4,4’-dimethoxybenzophenone (**6.2**) in 74% yield after isolation using 5 mol % RFTA under air (24 h), all performed without any additional solvent (Scheme 6). The authors noted that the reaction proceeds via a molten state, since in a control experiment without RFTA the mixture was found to liquefy. This was due to the heat generated by LEDs and not by the grinding action. Thus, light irradiation fulfils a double function: the excitation of the photocatalyst and melting of the substrate, inducing mobility of the molecules and the occurrence of the catalytic cycle. On a different note, the authors stressed that ball milling did not allow to form the expected product. This happened because light was unable to reach the photocatalyst in the latter setup, due to small amounts of solid adhering to the inner surface of the jar and the shielding effect within the milling chamber.

### Ball milling

Manual and vortex grinding present certain advantages, such as a low initial investment for conducting photomechanochemistry. However, these methods face significant limitations that restrict the integration of photomechanochemistry into synthetic workflows, both in academia and industry. They are inherently low in throughput, lack standardized equipment for photochemical reactions, and suffer from poor reproducibility. In fact, it is striking that often little detail is given regarding the geometry of the setup as well as the milling conditions. Additionally, these methods are neither scalable nor typically suitable for automation. Therefore, it comes as no surprise that the examples described above bear little synthetic potential.

The substantial technological advancements within the chemical field have impacted mechanochemistry as well, leading to the introduction of ball milling, which enables precise control over energy input, operating temperatures, and offers the potential for automated, reproducible processes. As an added benefit, the use of closed reactors ensures maximum operational safety, preventing direct exposure of the operator to potentially hazardous solid mixtures. A key technological challenge in this domain is to develop methods to effectively deliver photons to the reaction mixture.

**Scheme 7:**
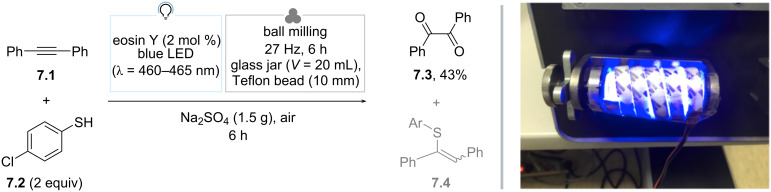
Photomechanochemical oxidation of 1,2-diphenylethyne to benzil. The photo in Scheme 7 was republished with permission of The Royal Society of Chemistry, from [70] (“Mechanochemically-assisted solid-state photocatalysis (MASSPC)” by V. Štrukil et al., Chem. Commun., vol. 53, © 2017); permission conveyed through Copyright Clearance Center, Inc. This content is not subject to CC BY 4.0

In 2017, Štrukil reported the first instance of a photochemical reaction conducted within a ball milling apparatus: the authors termed this approach “mechanochemically-assisted solid-state photocatalysis” (Scheme 7) [70]. Therein, a custom-built photoreactor was integrated into a standard laboratory ball mill apparatus. This reactor, equipped with blue LEDs, allowed for adjustable light intensity and maintained optimal temperature regulation using a fan. To overcome the challenge posed by the non-transparency of milling jars, a glass capsule was utilized. Teflon balls were employed to grind the reagents, thereby minimizing wear on the milling vessel. As a benchmark reaction, they selected the photochemical oxidation of 1,2-diphenylethyne to benzil [71]. Thus, when a 1:2 mixture of **7.1** and 4-chlorothiophenol (**7.2**) was ground in the presence of eosin Y as the photocatalyst and anhydrous Na_2_SO_4_ as a bulking agent, benzil (**7.3**) was obtained in 43% GC yield under blue light irradiation (LED, 14.5 W). Isolation led to diminished yields (ca. 35%). When milling was conducted in the dark, only traces of **7.3** were detected and an isomeric mixture of 1-(4-chlorophenylthio)stilbene (**7.4**, *E*:*Z* 33:67) was observed. The authors demonstrated that, when **7.4** (mixture) was irradiated, it was readily converted to **7.3** (36%, 4 h). Overall, it was concluded that **7.1** and **7.2** first react to give **7.4** via a dark mechanochemical thiol–yne reaction; the latter is then converted to **7.3** by singlet oxygen generated in situ, by eosin Y. It is important to notice here that the reaction is proposed to proceed according to a different mechanistic scenario than that operating in solution [71], where photogenerated thiyl radicals proved crucial intermediates.

**Scheme 8:**
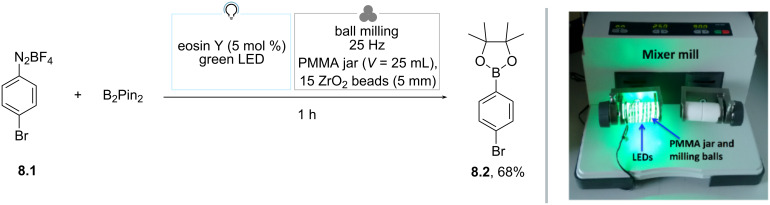
Photomechanochemical borylation of aryldiazonium salts. The photo in Scheme 8 was reproduced from [72] (© 2017 J. G. Hernández et al., published by Beilstein-Institut, distributed under the terms of the Creative Commons Attribution 4.0 International License, https://creativecommons.org/licenses/by/4.0).

Parallelly, Hernández reported a photomechanochemical approach for the borylation of aryldiazonium salts in a ball milling apparatus equipped with a transparent polymethylmethacrylate (PMMA) jar (Scheme 8) [72]. As far as the irradiation source is concerned, the authors wrapped a LED strip around the jar. A solution-based precedent by the Yan group was used as a benchmark transformation [73]. Under optimized conditions, a mixture of diazonium salt **8.1**, B_2_Pin_2_ (1.5 equiv) and eosin Y (5 mol %) was irradiated with green light while being milled in a 25 mL PMMA milling jar with 15 ZrO_2_ balls of 5 mm in diameter at 25 Hz. The expected product **8.2** was obtained in 68% yield after isolation. A comprehensive set of control experiments highlighted the essential role of mechanochemistry in enhancing the mixing efficiency and increasing the exposure of the surface of the reaction mixture to light. Additionally, the formation of compound **8.2** was associated with the observation of an initial molten state of the mixture, which could have promoted single-electron-transfer processes that are crucial for product formation. Lastly, the author noted that the inclusion of acetonitrile as a LAG agent enabled the transformation even without the presence of a photocatalyst [72,74].

**Scheme 9:**
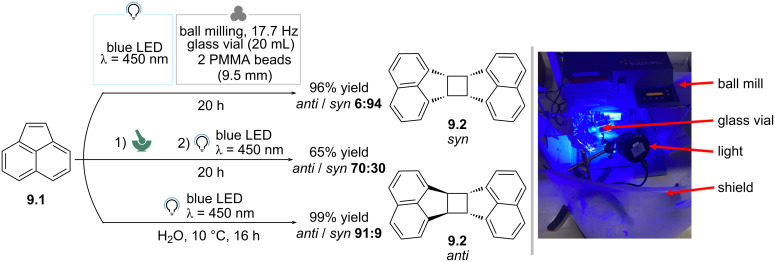
Photomechanochemical control over stereoselectivity in the [2 + 2] dimerization of acenaphthylene. The photo in Scheme 9 was republished with permission of The Royal Society of Chemistry, from [75] (“Photomechanochemical control over stereoselectivity in the [2 + 2] photodimerization of acenaphthylene” by S. Biswas et al., Faraday Discuss., vol. 241, © 2023); permission conveyed through Copyright Clearance Center, Inc. This content is not subject to CC BY 4.0.

In 2023, Braunschweig and co-workers reported the photomechanochemical [2 + 2] cycloaddition of acenaphthylene (**9.1**) (Scheme 9) [75]. In this case the authors used a ball-mill reactor equipped with a blue LED, and the reaction was run in a glass vial with two transparent PMMA balls. The authors noted that fluorination of the vial and the use of silica gel to adjust texture were needed to prevent reagent adhesion on the vial walls. Thus, the reactor was operated at 17.7 Hz for 20 h and the dimerization to **9.2** occurred in 96% ^1^H NMR yield with a 6:94 *anti*/*syn* selectivity. A different selectivity was observed when crystals were first ground, and then irradiated under argon without applying forces (65% yield, 70:30 *anti*/*syn*). This indicates a unique divergent photomechanochemical reaction pathway with respect to the simple irradiation in solid state. Remarkably, no selectivity was observed in solution state (DMSO as solvent), while a completely reversed selectivity (99%, *anti*/*syn* 91:9) was obtained when using an anti-solvent (H_2_O). To account for the unique outcome of photomechanochemical conditions, the authors performed a DFT study and introduced the concept of mechanosusceptibility. Briefly, they computationally applied a force along the C–C-bond-forming tensor that would result in the formation of either the *syn* or the *anti* products, and found that the bond-forming event leading to the former can occur at lower applied forces.

**Scheme 10:**
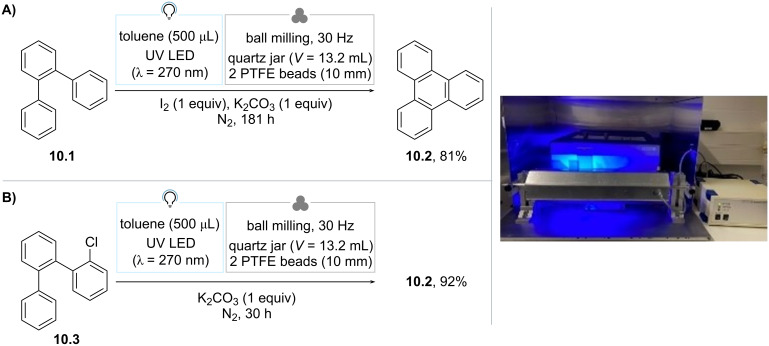
Photomechanochemical synthesis of polyaromatic compounds using UV light. The photo in Scheme 10 was reproduced from [76] (© 2023 D. M. Baier et al., published by Wiley-VCH GmbH, distributed under the terms of the Creative Commons Attribution-NonCommercial 4.0 International License, https://creativecommons.org/licenses/by-nc/4.0/). This content is not subject to CC BY 4.0.

In the same year, Borchardt reported the first use of a ball mill with UV light for Mallory and cyclodehydrochlorination reactions (Scheme 10) [76]. The authors proposed a photomechanochemical reactor where the jar of the ball mill vibrates within an aluminum frame on which UV-C LEDs are mounted. The milling vessel used in this process is a cylinder of quartz glass with PFA (perfluoroalkoxyalkane) caps on both ends to absorb impacts. Thus, the Mallory reaction to get triphenylene was investigated. When *o*-terphenyl (**10.1**) was milled (30 Hz, PTFE balls) and irradiated (λ = 270 nm) in the presence of silica gel (bulking agent), I_2_ (1 equiv) as an oxidant, and K_2_CO_3_ (1 equiv) as a base in the presence of toluene, the expected product **10.2** was obtained in 81% yield after isolation upon 181 h of irradiation. The authors claim that toluene acts as a photosensitizer since cyclohexane, which has similar solubilizing power, did not serve well as a LAG agent. The authors demonstrated that nanographenes could be obtained via cyclodehydrochlorination of **10.3** under photomechanochemical conditions as well. Also in this case, the addition of toluene was beneficial and excellent yield of the corresponding product (**10.2**) was observed in 30 h of reaction. As a comparison, the latter reaction required 48 h in solution. Intriguingly, **10.3** could in turn be synthesized via a mechanochemical Suzuki coupling, demonstrating superior performance compared to the solution-phase approach. This mechanochemical method afforded **10.3** in approximately the same yield, but significantly faster, completing the reaction in 2 h instead of 24 h. Overall, in this case, milling ensures the necessary mixing of the reactants and continuously exposes fresh surfaces of the solids to light. The authors were able to scale the conversion of **10.3** to **10.2** on a gram scale (1 g), even though with a diminished yield (48%) due to inefficient mixing.

**Scheme 11:**
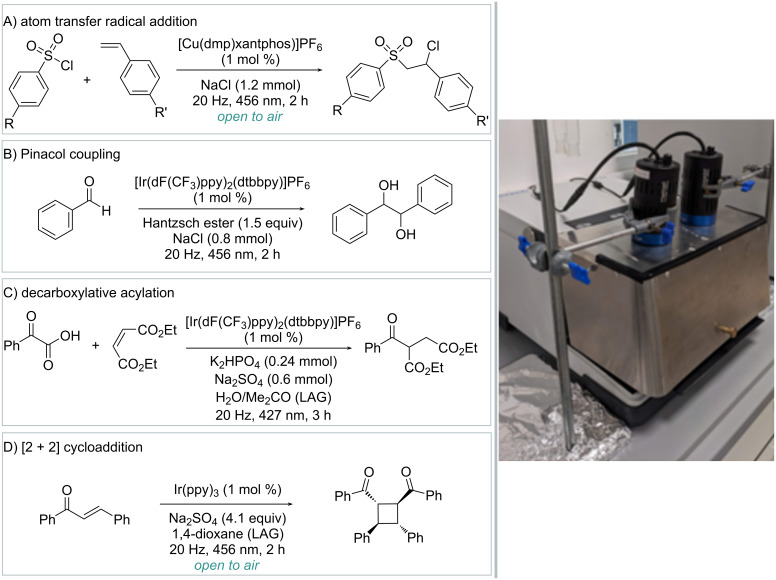
Mechanically assisted photocatalytic reactions: A) atom-transfer-radical addition, B) pinacol coupling, C) decarboxylative alkylation, D) [2 + 2] cycloaddition. The photo in Scheme 11 was reproduced from [77] (© 2024 F. Millward et al., published by Wiley-VCH GmbH, distributed under the terms of the Creative Commons Attribution 4.0 International License, https://creativecommons.org/licenses/by/4.0).

In a recent instance, Millward and Zysman-Colman reported a comprehensive exploration of the benefits of the photomechanochemical approach in the field of synthesis [77]. Specifically, they developed photomechanochemical conditions for the atom-transfer-radical addition (ATRA) of sulfonyl chlorides to alkenes, pinacol coupling of carbonyl compounds, decarboxylative acylation, and photocatalyzed [2 + 2] cycloaddition (Scheme 11).

Regarding the experimental setup, the authors adapted a commercially available ball mill, by introducing a custom-made stainless steel safety shield to minimize unwanted light leakage. The interior surface of the shield is somewhat reflective. A hole in the milling shield above each jar holder allows for irradiation of the reaction glass vessels from above using powerful LED lamps.

First, photomechanochemistry facilitated the copper-photocatalyzed ATRA of sulfonyl chlorides and styrenes under fully aerobic conditions (Scheme 11A), a feature unattainable in traditional solution-phase synthesis. In fact, in the latter case, almost complete recovery of the starting material was observed. Second, the authors found that the replacement of diisopropylethylamine (DIPEA) with Hantzsch ester in the role of sacrificial reductant for the pinacol coupling of benzaldehyde enables solvent-free conditions for this transformation (Scheme 11B). Here, the aggregation state of the substrate and the reductant proved to be crucial to establish a fully operative protocol. The third reaction tested under mechanophotocatalytic conditions was the well-established decarboxylative acylation of electrophilic olefins (Scheme 11C). Compared to solution-phase photocatalysis, photomechanochemical conditions allowed to reduce the reaction time from 24 h to 3 h while maintaining comparable yields. Finally, the authors proved that photomechanochemistry contributes to the initial rate enhancement of photocatalyzed [2 + 2] cycloadditions under aerobic conditions, even though over longer irradiation times the solution-state yields are superior (Scheme 11D).

**Scheme 12:**
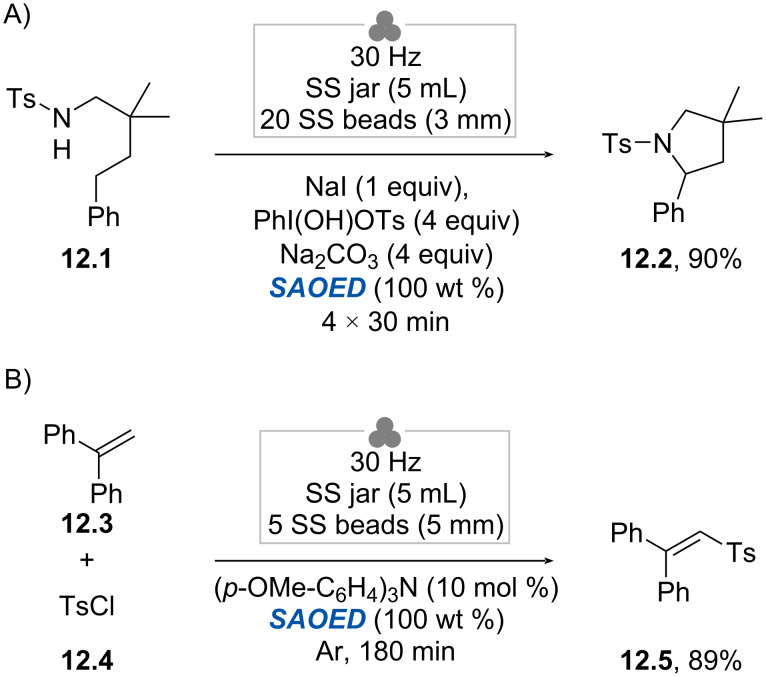
Use of mechanoluminescent materials as photon sources for photomechanochemistry. SAOED: SrAl_2_O_4_:Eu^2+^/Dy^3+^. A) Hofmann–Loffler–Freytag reaction; B) EDA-based photochemistry [78].

Very recently, Wu, Wang, and co-workers proposed a radically different approach to photomechanochemistry [78]. Rather than trying to interface two technologies, the authors opted to use mechanoluminescent powders to generate photons directly inside the jar. In more detail, SrAl_2_O_4_:Eu^2+^/Dy^3+^ (SAOED) was used as a mechanoluminescent material to drive the visible-light-mediated Hofmann–Loffler–Freytag (HLF) reaction (Scheme 12A). Thus, when using a mixture of **12.1** (0.2 mmol), NaI (1 equiv), Koser’s reagent, and Na_2_CO_3_ (each 4 equiv), in the presence of 100 wt % of SAOED, pyrrolidine **12.2** was isolated in 90% yield. As far as the mechanochemical setup is concerned, the authors milled the reaction mixture at 30 Hz in a 5 mL stainless-steel jar equipped with twenty stainless-steel balls (Ø = 3 mm). Intriguingly, the authors noticed that multiple smaller balls, when used in a comparable total mass, outperformed a single larger ball in terms of reaction efficiency. Moreover, intermittent milling (4 × 30 min periods with 5 min breaks instead of 2 h in a row) proved beneficial to minimize heat accumulation resulting in product decomposition. Next, the authors proved the generality of mechanoluminescence by performing the sulfonylation of alkenes via EDA-complexes photochemistry (Scheme 12B). Thus, when a mixture of 1,1-diphenylethylene (**12.3**, 0.2 mmol), tosyl chloride (**12.4**, 2 equiv), and tris(4-methoxyphenyl)amine (10 mol %) was milled in the presence of 100 wt % of SAOED at 30 Hz for 3 h, sulfone **12.5** was obtained in 89% yield after isolation. The synthesis of **12.5** was scaled up to 15 mmol scale, obtaining the desired product in a yield of 79%. Scanning electron microscopy (SEM) analysis revealed the deformation and reduction in size of SAOED particles upon milling. Interestingly, carbon determination experiments revealed that SAOED is quickly poisoned by organic matter, thus hampering recyclability. This can be to some extent reversed via calcination (700 °C, 2 h).

## Conclusion and Future Directions

In this Perspective, we have examined how photons and impact forces have been merged so far. Although early examples of photomechanochemistry date back to the 1980s, there has been a recent surge in interest. It is important to note that photomechanochemistry remains in its early stages, with studies thus far focusing primarily on straightforward transformations, such as [2 + 2] photodimerization, and a limited number of preparative examples. In most examples, mechanochemistry is proposed to constantly expose fresh surface to light and promote mass transfer. As understanding of the methodology advances, the development of more complex synthetic strategies, including C–H, C–C, and C–heteroatom-bond formation, is expected [79]. Moreover, we anticipate that the difference in photophysical properties between organic molecules in diluted and extremely concentrated solutions will lead to unprecedented reactivity.

From a conceptual standpoint, the examples presented above effectively illustrate that each discipline offers unique benefits within this marriage. On one side, as amply discussed in the introduction, photochemistry allows to leverage reactivity modes inaccessible through traditional approaches. On the other side, mechanochemistry brings around advantages in terms of sustainability and operational efficiency. Firstly, it either eliminates the need for solvents entirely or significantly reduces their use, as in liquid-assisted grinding (LAG). This minimizes waste production compared to conventional solution-based methods. This is particularly advantageous when high-boiling point (e.g., DMSO) or noxious solvents (e.g., DMF and halogenated ones) are required. It is worth noting, however, that in some cases the heat generated from the high-power light sources causes the reaction mixture to melt, which might not be general, but rather substrate-dependent. Secondly, mechanochemistry allows the synthesis practitioner to run reactions at maximal concentrations, accelerating reaction rates and enabling processes involving insoluble reagents and/or photocatalysts. Additionally, as discussed, the use of impact forces can lead to unique selectivity profiles compared to solution-based methods, further enhancing its utility. Thirdly, dissolved oxygen must be often meticulously removed in solution-based methods via tedious techniques such as freeze-pump-thaw. By eliminating solvents, mechanochemistry enables the development of more practical and efficient photochemical protocols, especially at scale, where degassing large volumes is technologically and economically challenging.

From a practical standpoint, the progression from manual grinding to ball milling was a natural development, yet there is still significant potential for further advancements. The examples discussed above clearly show that mechanochemistry and light-driven synthesis have evolved at different rates, with each field capturing the interest of distinct research communities. This has led to the creation of hybrid systems – improvised combinations of commercial ball mills with transparent jars (e.g., PMMA, glass, quartz, or epoxy resin) and off-the-shelf LED lamps or strips. However, a standardized apparatus for photomechanochemistry is not available yet. Looking forward, designing specialized equipment capable of integrating both mechanical impact forces and photon input would be highly beneficial.

Moreover, a major challenge limiting the widespread adoption of mechanochemistry in industrial applications is scalability [80], a concern that also extends to photomechanochemistry. One potential solution to address this issue has been the introduction of twin-screw extruders [81]. However, it remains uncertain whether this approach can be effectively adapted for use in photomechanochemical processes. Such dedicated machinery could enable a synergistic interaction between photons and forces, streamlining the combination of mechanochemical and photochemical processes and paving the way for more efficient, sustainable, and selective transformations in organic synthesis. An unexplored opportunity is offered by resonant acoustic mixing (RAM) [82], a technology that leverages low frequency acoustic waves to deliver controlled mechanical force within a reaction vessel. In this case, cheap commercially available glass vessels present in all laboratories can be used, thus solving the issue of fabricating custom-made transparent jars. Intriguingly, RAM appears an ideal technique for high-throughput experimentation [83]. A further area of development would be the possibility of integrating options for the in-situ monitoring of reactions, such as X-ray and Raman techniques [47–48,84–87].

**Figure 3:**
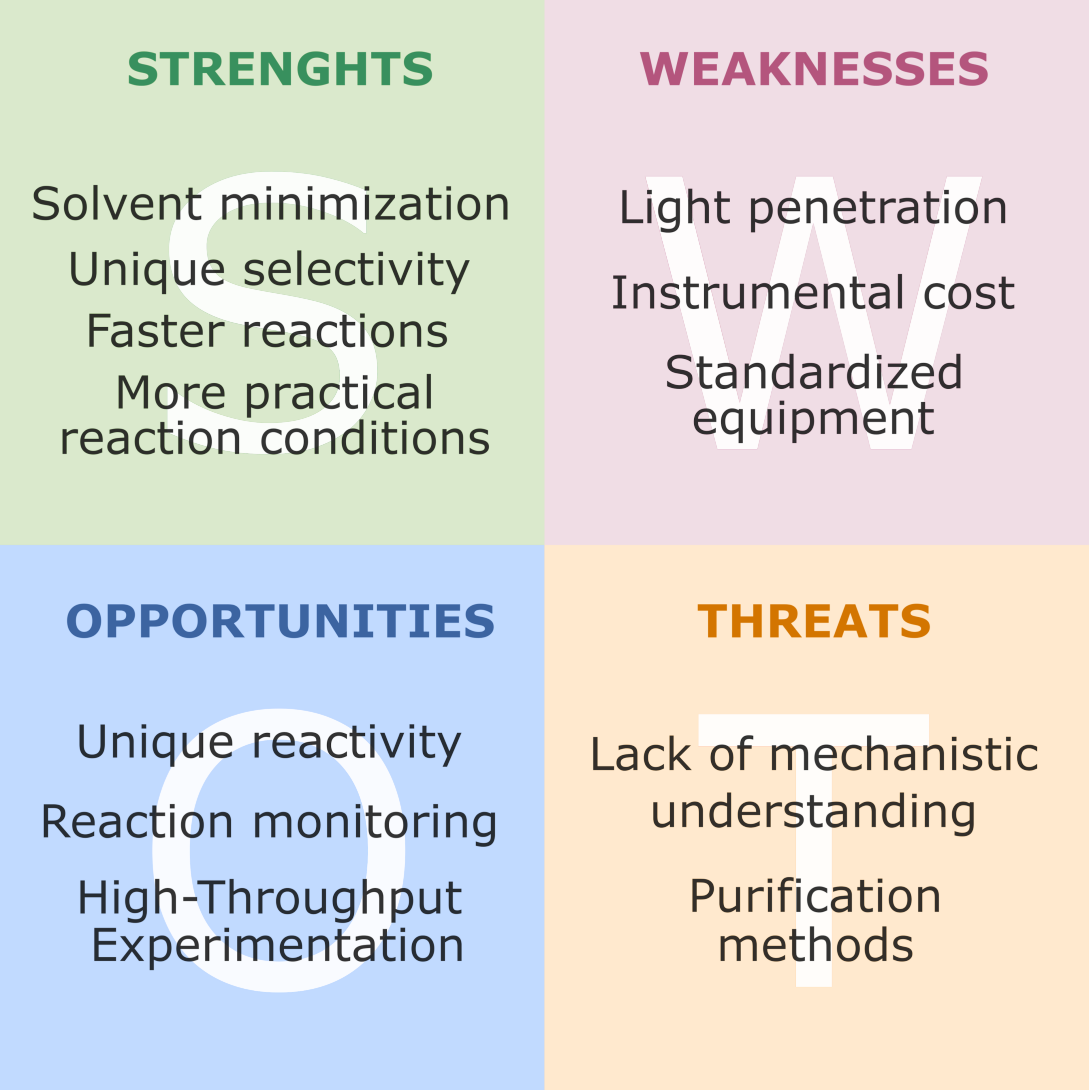
SWOT (strengths, weaknesses, opportunities, threats) analysis of photomechanochemistry.

In conclusion, in recognizing that both photochemistry and mechanochemistry provide highly sustainable approaches for synthesis, we envision a powerful synergy between these fields. In fact, we provide a SWOT (*Strengths, Weaknesses, Opportunities, Threats*) analysis for the strategic development of this new exciting field (Figure 3). By combining their knowledge, researchers working at this interface have the potential to redefine the landscape of sustainable synthesis, with this Perspective serving as a snapshot of the current state-of-the-art.

## Data Availability

Data sharing is not applicable as no new data was generated or analyzed in this study.
